# Quantification of myocardial ischemia and subtended myocardial mass at adenosine stress cardiac computed tomography: a feasibility study

**DOI:** 10.1007/s10554-021-02314-z

**Published:** 2021-06-23

**Authors:** F. Y. van Driest, R. J. van der Geest, A. Broersen, J. Dijkstra, M. el Mahdiui, J. W. Jukema, A. J. H. A. Scholte

**Affiliations:** 1 Department of Cardiology, Leiden Heart-Lung Center, Leiden University Medical Center, Albinusdreef 2, 2333 ZA Leiden, Netherlands; 2 Department of Radiology, Division of Image Processing, Leiden University Medical Center, Albinusdreef 2, 2333 ZA Leiden, Netherlands

**Keywords:** Coronary computed tomography angiography, Cardiac ct perfusion imaging, Ischemia, Algorithms

## Abstract

Combination of coronary computed tomography angiography (CCTA) and adenosine stress CT myocardial perfusion (CTP) allows for coronary artery lesion assessment as well as myocardial ischemia. However, myocardial ischemia on CTP is nowadays assessed semi-quantitatively by visual analysis. The aim of this study was to fully quantify myocardial ischemia and the subtended myocardial mass on CTP. We included 33 patients referred for a combined CCTA and adenosine stress CTP protocol, with good or excellent imaging quality on CTP. The coronary artery tree was automatically extracted from the CCTA and the relevant coronary artery lesions with a significant stenosis (≥ 50%) were manually defined using dedicated software. Secondly, epicardial and endocardial contours along with CT perfusion deficits were semi-automatically defined in short-axis reformatted images using MASS software. A Voronoi-based segmentation algorithm was used to quantify the subtended myocardial mass, distal from each relevant coronary artery lesion. Perfusion defect and subtended myocardial mass were spatially registered to the CTA. Finally, the subtended myocardial mass per lesion, total subtended myocardial mass and perfusion defect mass (per lesion) were measured. Voronoi-based segmentation was successful in all cases. We assessed a total of 64 relevant coronary artery lesions. Average values for left ventricular mass, total subtended mass and perfusion defect mass were 118, 69 and 7 g respectively. In 19/33 patients (58%) the total perfusion defect mass could be distributed over the relevant coronary artery lesion(s). Quantification of myocardial ischemia and subtended myocardial mass seem feasible at adenosine stress CTP and allows to quantitatively correlate coronary artery lesions to corresponding areas of myocardial hypoperfusion at CCTA and adenosine stress CTP.

## Introduction

In patients with coronary artery disease (CAD) an imaging protocol combining coronary computed tomography angiography (CCTA) and adenosine stress CT myocardial perfusion (CTP) allows for anatomical and functional assessment of coronary artery lesions as well as myocardial ischemia [1, 2]. The decision to revascularize patients depends both on the lesion severity and location as well as the extent of the relative hypoperfused (ischemic) myocardium, relative to the subtended myocardial mass distal of the coronary stenosis [1]. However, adenosine stress CTP is nowadays assessed semi-quantitatively by visual analysis on a routine basis in many centres.

The Voronoi algorithm is a mathematical algorithm that enables users to divide a two-dimensional area or three-dimensional space by predetermined points based on the shortest distance to those points. This algorithm can be used to divide tissue supplied by different blood vessels according to which blood vessel is closest to the tissue. By using a Voronoi-based segmentation algorithm on myocardial tissue it seems possible to quantify the subtended myocardial mass for each lesion in the coronary tree [3]. By also quantifying the hypoperfused myocardium itself we aim to identify the distribution of myocardial ischemia over the coronary artery lesion(s). To the best of our knowledge this has never been done in a fully quantitative manner for adenosine stress CTP. Therefore, we hypothesize that full quantification of adenosine stress myocardial ischemia and subtended myocardial mass using this Voronoi-based segmentation algorithm is feasible and may ease detection of hemodynamically significant lesions.

## Materials and methods

### Patients

33 patients with chest pain complaints, referred for a combined CCTA and adenosine stress CTP protocol were included in the current study. As manual drawing of perfusion defects is dependent on scan quality of adenosine stress CTP, only patients with good or excellent imaging quality of these scans were selected from our CTP database containing 241 patients. Patients with normal CTP images or fixed perfusion defects were excluded because reversible ischemia is or may be absent in these cases, respectively [4]. Clinically acquired data were retrospectively analysed. The institutional review board of the Leiden University Medical Center, The Netherlands, approved this retrospective evaluation of clinically collected data and waived the need for written informed consent.

### Data acquisition and analysis

CCTA and static adenosine stress CTP were performed using a 320-row volumetric scanner (Aquilion ONE, Canon Medical Systems and Aquilion ONE Genesis Edition, Canon Medical Systems, Otawara, Japan). Consumption of caffeine products 24 h before examination was discouraged. One hour before CCTA heart rate and blood pressure were monitored. If a patient’s heart rate exceeded 60 beats per minutes (bpm) and no contraindications were present metoprolol, 25 mg up until 150 mg, was administrated orally. If the heart rate remained above 60 bpm additional metoprolol was injected intravenously.

Prior to CCTA nitroglycerin (0.4 mg) was administered sublingually. Scan parameters for CCTA were as follows: peak tube voltage 100–135 kV with a tube current of 140-580mA. A detector collimation of 320 × 0.5 mm, a 275 ms gantry rotation time and temporal resolution of 137 ms for the Aquilion ONE Genesis Edition and a detector collimation of 320 × 0.5 mm, 350 ms gantry rotation time and temporal resolution of 175 ms for the Aquilion ONE. Prospectively electrocardiogram (ECG) triggering was used to scan 70–80% of the RR interval. When heart rate was above 65 bpm 30–80% of the RR-interval was scanned. First 50 to 90 ml of contrast agent (Iomeron 400, Bracco, Milan, Italy) was administered in the antecubital vein. Hereafter, a 20 mL of a 1:1 mixture of contrast and saline and finally 25 mL of saline was administered. CCTA was performed the next beat when the threshold of 300 Hounsfield units (HU) was reached in the descending aorta.

In patients with suspicion of significant stenosis (≥ 50%) at CCTA, adenosine stress CTP was performed at least 20 min after CCTA. Blood pressure and electrocardiogram were monitored during 4 min of continuous adenosine infusion (0,14 mg/kg/min) after which a contrast agent was administered. After reaching a target threshold of 300 HU in the descending aorta CTP images were acquired the next heartbeat scanning 80–99% of the RR interval. Contrast agent, injection protocol and tube settings were all similar to the CCTA acquisition.

### Data processing

**Fig. 1:**
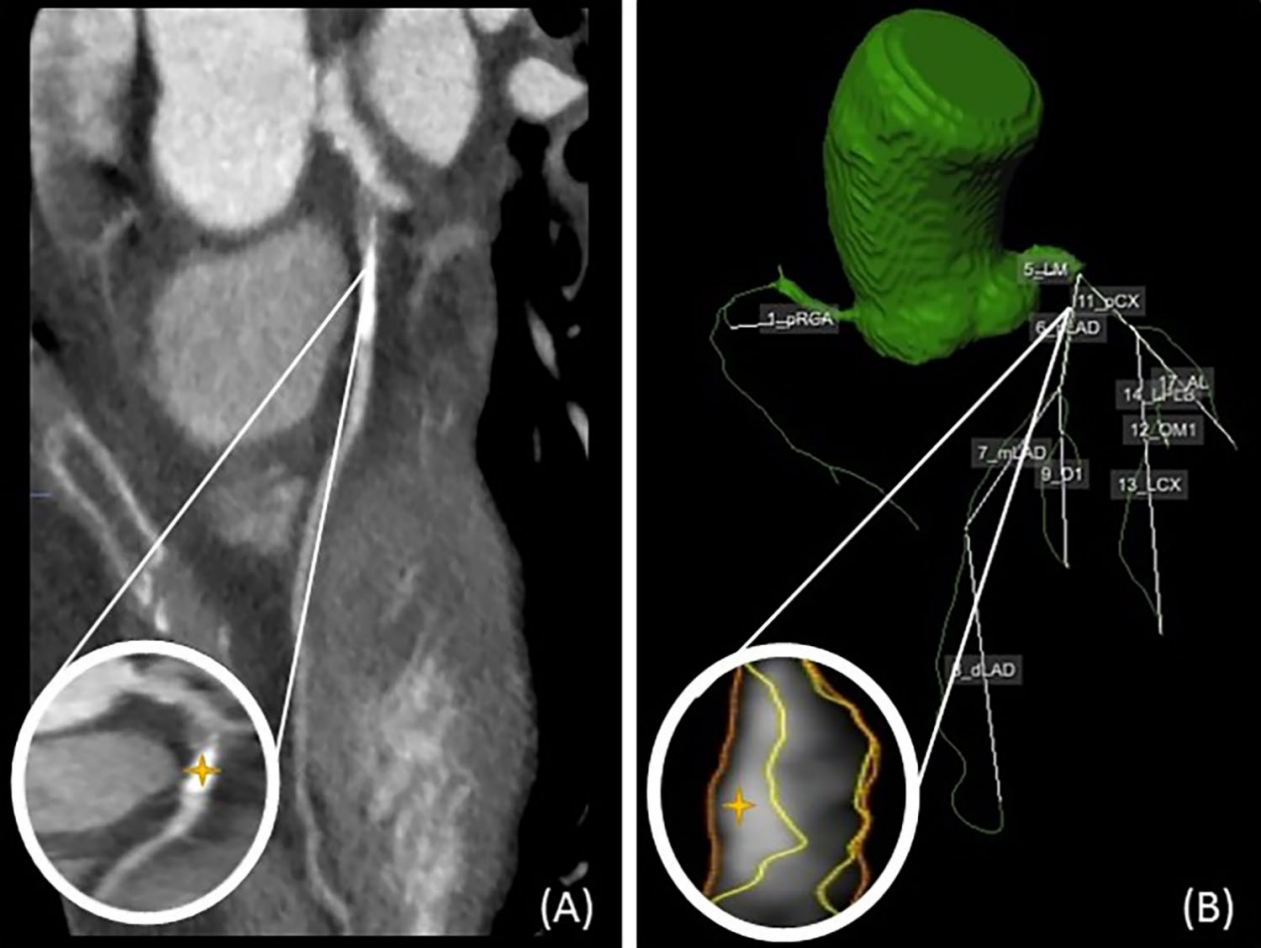
Resting CCTA is used for automatically extracting the main branches of the coronary artery tree (**B**). A lesion is shown in the proximal left anterior descending (LAD) and marked by the yellow star (**A**). Subsequently, we can define the proximal LAD stenosis marked by the yellow star (**B**)

Images were transferred to a workstation and the main branches of the coronary artery tree were automatically extracted from the CCTA. Assessment of the CCTA was done by trained cardiologists with at least 10 years of experience. A luminal stenosis of ≥ 50% was considered significant. Proximal and distal part of the relevant lesion were manually defined using dedicated software (QAngio CT Research Edition v3.1.5.1 Medis Medical Imaging, Leiden, The Netherlands) (Fig. 1). If one vessel, or its side branches had multiple relevant coronary artery lesions we defined only the most proximal one. The most proximal part of the lesion was used as the starting point for calculating the subtended mass.

**Fig. 2:**
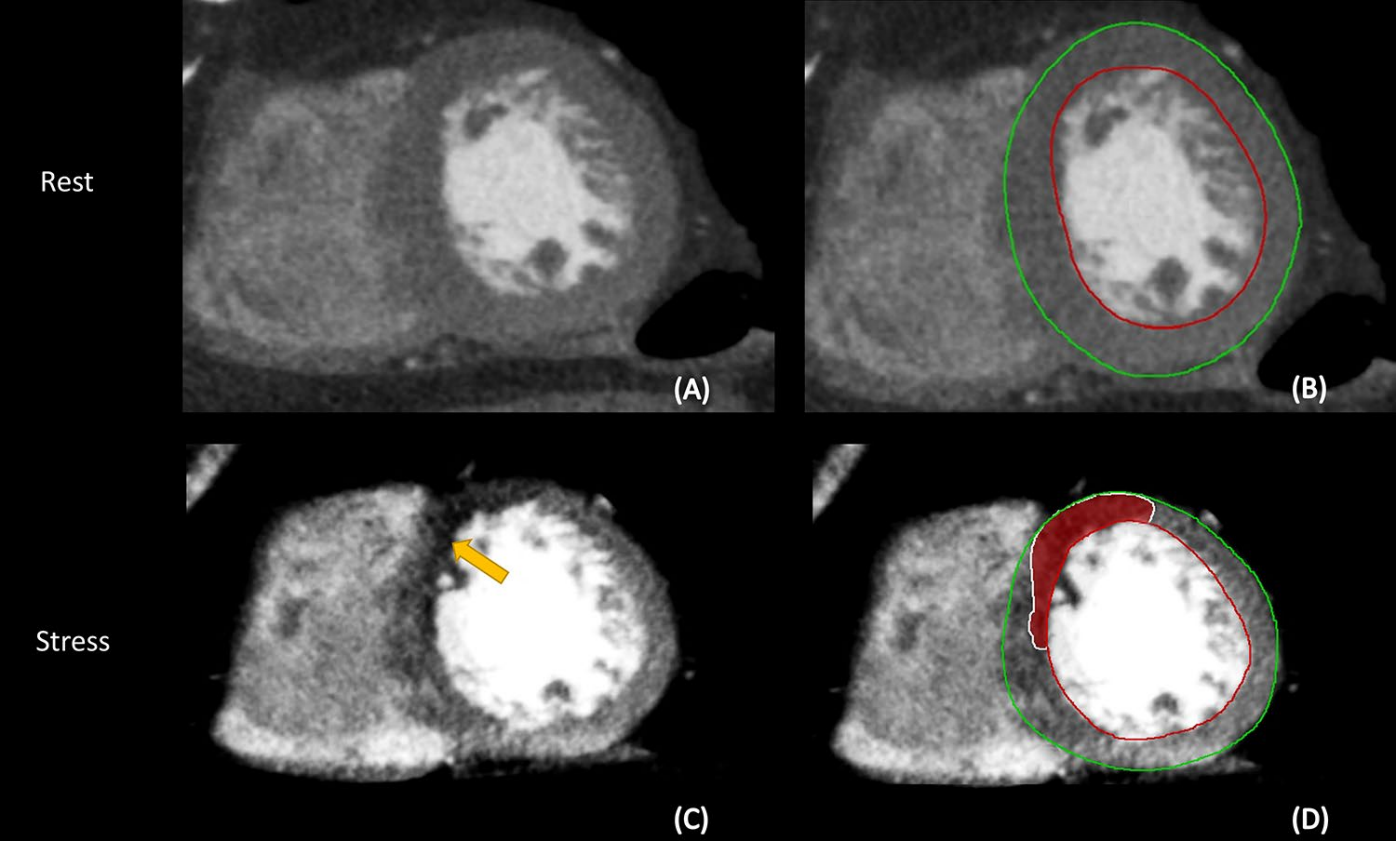
Left ventricular epicardial and endocardial contours were semi-automatically defined in short-axis reformatted images in both the resting CCTA (**A**, **B**) and the adenosine stress CTP (**C**, **D**). A perfusion defect is marked by the yellow arrow (**C**). The perfusion defect is manually drawn in the short axis reformatted images from the adenosine stress CTP (**D**)

Further processing of the images was performed using in-house developed MASS software (Leiden University Medical Centre). The CCTA and adenosine stress CTP image data were manually reformatted into a short-axis orientation covering the complete left ventricle with an inter-slice spacing of 4 mm. Subsequently, left ventricular epicardial and endocardial contours were semi-automatically defined in both the CCTA and the adenosine stress CTP images. Using a narrow window width and level setting (W300/L150) and a slice thickness of 4 mm, perfusion defects were manually drawn in the short axis slices derived from the CTP scan (Fig. 2). Registration was performed to spatially align the CCTA and CTP images and results of image segmentation were exported as 3D objects in VTK format for further analysis and visualization. The software uses the epicardial and endocardial contours from the CCTA for automatically calculating the left ventricular mass.

To assess reproducibility two observers (F.D. and I.H) were blinded to the original contours and a sample of ten cases was randomly selected in which left ventricular epicardial and endocardial contours were again semi-automatically defined and subtended mass was recalculated using the Voronoi-based algorithm. Also, perfusion defects were manually re-drawn and re-measured in grams. Correlations were subsequently tested between new and prior results concerning left ventricular mass, total subtended mass and perfusion defect mass with Pearson’s correlation coefficient using SPSS software (version 25, SPSS IBM Corp, Armonk, New York).

### Voronoi-based segmentation

**Fig. 3:**
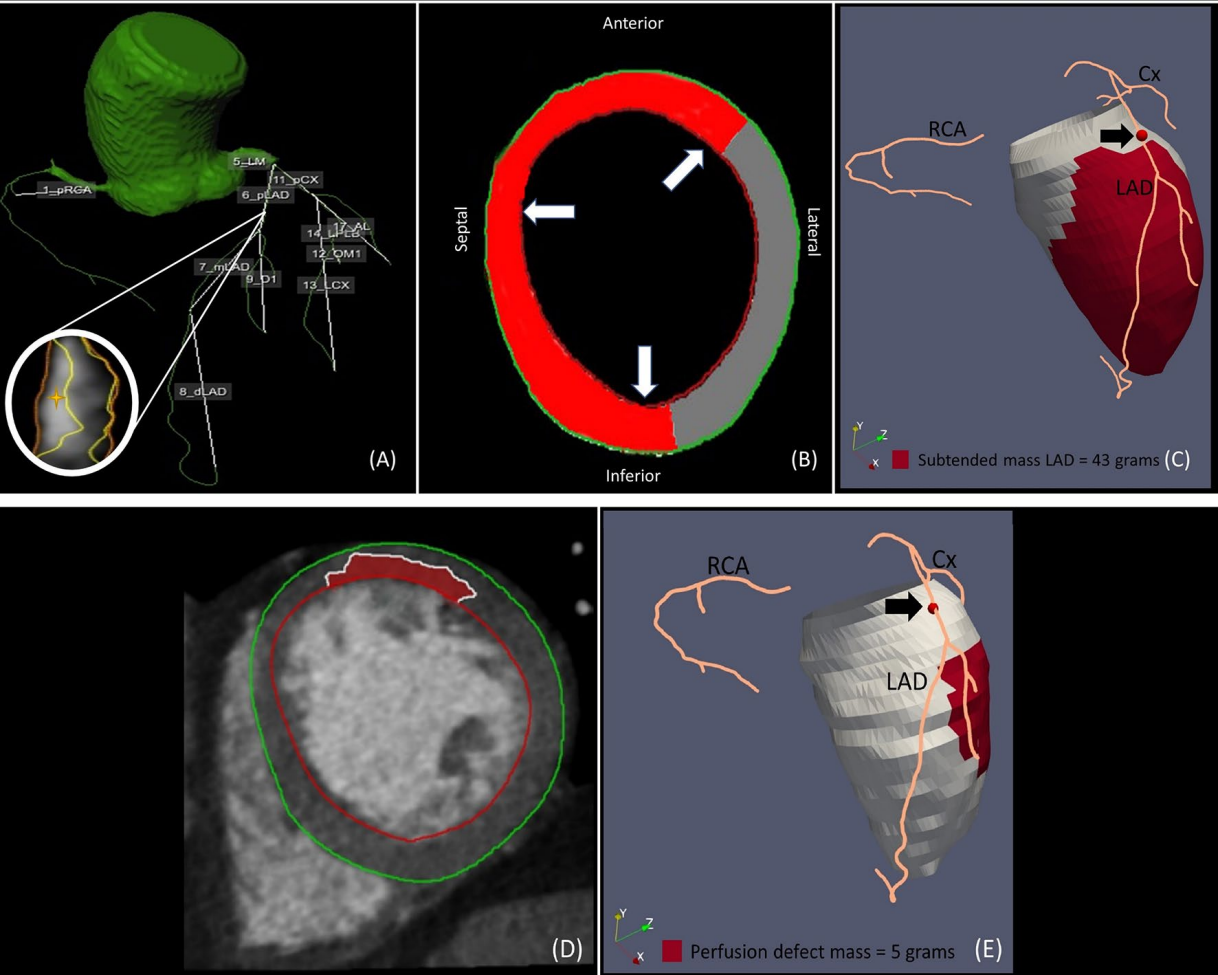
Segmented coronary artery tree and identified relevant coronary artery lesion in the proximal LAD (**A**) are used for computing the subtended myocardial mass in red in the short-axis view using our Voronoi-based algorithm (**B**). This can be further visualized in 3D in which the red dot (marked by the black arrow) corresponds to the relevant coronary artery lesion and the red area corresponds to the subtended myocardium which is calculated by our Voronoi-based segmentation as 43 g (**C**). The manually drawn perfusion defect (**D**) is also visualized and quantified (**E**)

**Fig. 4:**
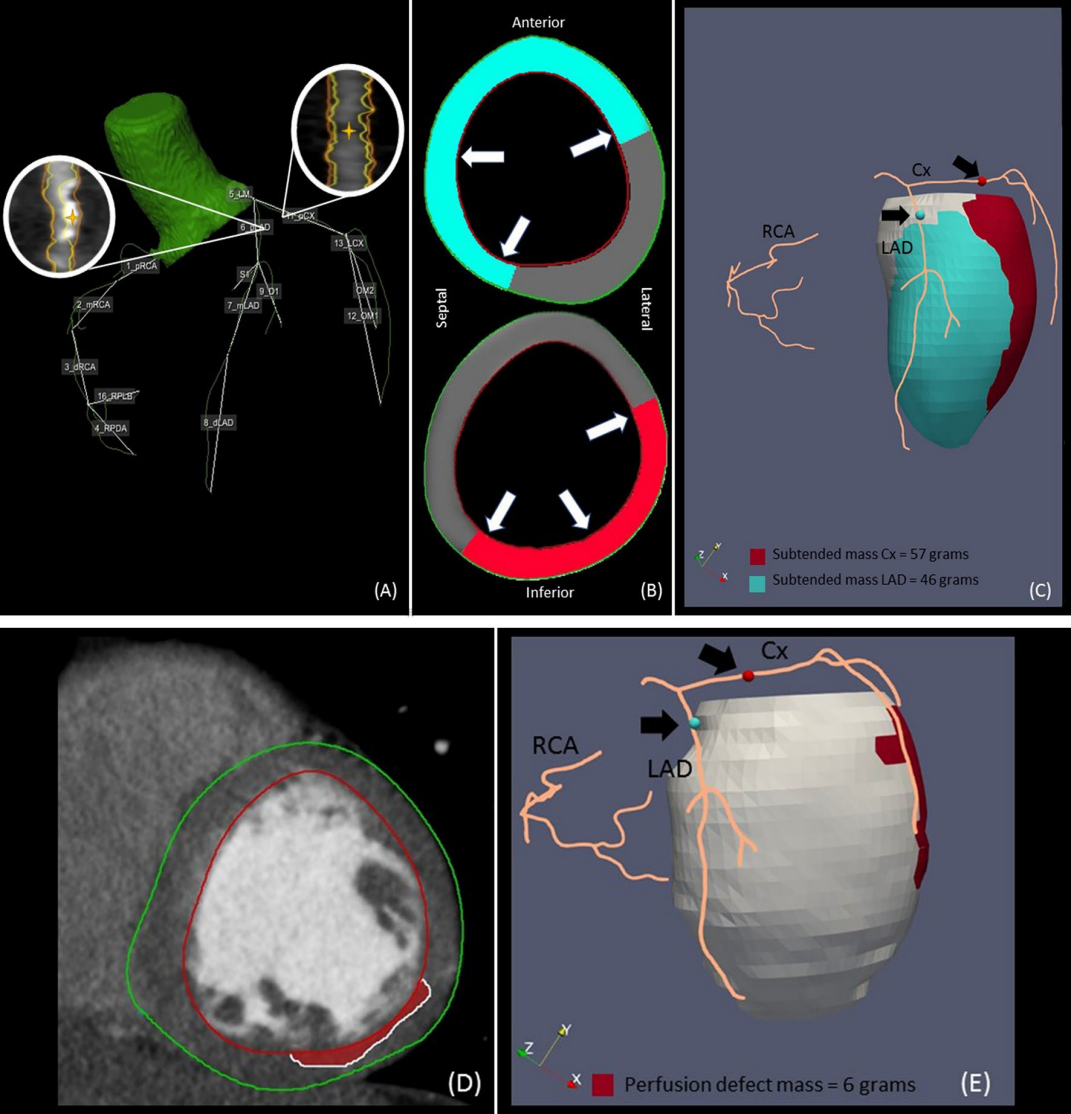
Coronary artery tree and defined relevant lesions in the proximal LAD and circumflex (Cx) (**A**) are used for computing the subtended myocardial mass in the short-axis view using our Voronoi based algorithm with in cyan the LAD lesion and the Cx lesion in red (**B**). This can be further visualized in 3D in which the red and cyan dots (marked by the black arrows) correspond to the relevant coronary artery lesions and the red and cyan area correspond to the subtended myocardium for that lesion. We calculated the subtended mass for the Cx lesion and LAD lesion as 57 and 46 g respectively (**C**). The manually drawn perfusion defect (**D**) is also visualised and measured (**E**)

A segmentation algorithm based on the Voronoi method was used on the CCTA in order to find the nearest location of the extracted coronary artery tree for every voxel within the left ventricular myocardium [3]. From this data the subtended myocardial mass could be computed, i.e. the left ventricular mass distal from a relevant coronary artery lesion (Fig. 3). Also, the perfusion defect was measured and visualised separately (Fig. 3). An example of a patient with multivessel disease is depicted in Fig. 4. Executing the algorithm for Voronoi based segmentation took approximately 1 min per lesion.

**Fig. 5:**
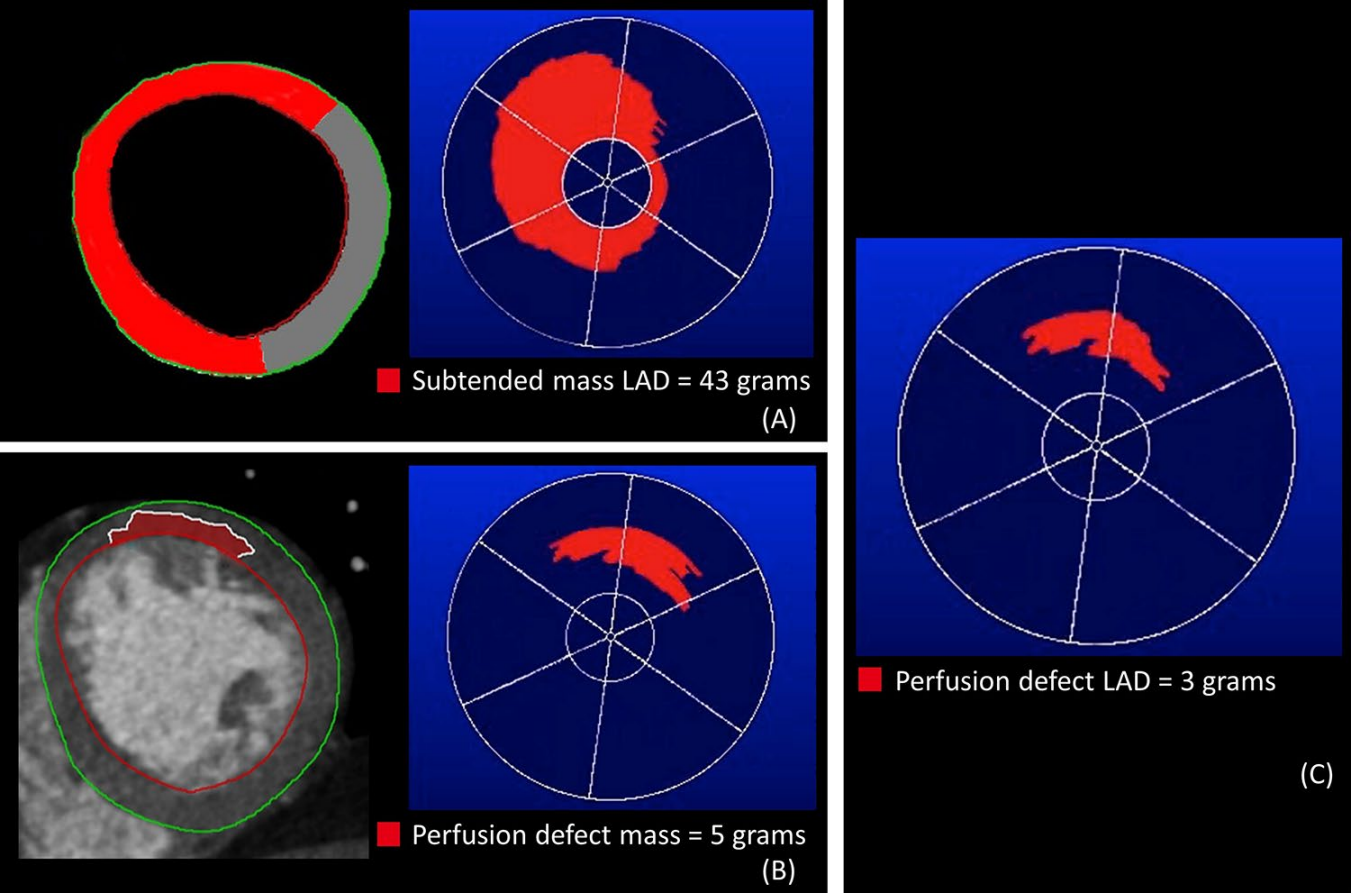
The subtended myocardial mass for one lesion pictured in red is measured in grams (**A**). The same is done for the perfusion defect (**B**). Perfusion defect per lesion is measured by calculating the intersection of A and B (**C**)

Finally, we quantified the subtended myocardial mass and perfusion defect mass per lesion using bullseye plots with MASS software. This process is depicted in Fig. 5. Figure 5A demonstrates the subtended myocardial mass-pictured in red- for one lesion calculated by using our Voronoi-based algorithm. Figure 5B represents the manually drawn perfusion defect. Subsequently, Fig. 5C represent the perfusion defect per lesion by calculating the intersection of figure A and B. For all measurements we used the endo- and epicardial contours from the resting CCTA. Subsequently, we calculated the total subtended mass. See formula:

$${\text{Total}}\,{\text{subtended}}\,{\text{mass}}\,{\text{ = }}\,{\text{Subtended}}\,{\text{mass}}\,{\text{lesion}}\,{\text{a}}\,{\text{ + }}\,{\text{Subtended}}\,{\text{mass}}\,{\text{lesion}}\,{\text{b}}$$

## Results

CCTA and adenosine stress CTP images from 33 patients (20 men, mean age, 67.8 ± 8.2 years) were used for analysis. Table 1 lists patient characteristics. We were able to successfully apply the Voronoi-based segmentation algorithm on all cases to quantify the subtended myocardial mass (per lesion) and perfusion defect mass (per lesion). Left ventricular mass was automatically calculated from the epicardial and endocardial contours with an average value of 118 g. We assessed a total of 64 relevant coronary artery lesions. Average values for total subtended mass, subtended mass per lesion, perfusion defect mass and perfusion defect mass per lesion were 69, 36, 7 and 3 g respectively. In 19/33 patients (58%) the total perfusion defect mass could be distributed over the relevant coronary artery lesion(s). Results were highly reproducible as demonstrated by respectively intra- and inter-observer correlation coefficients for left ventricular mass (r = 0,970 and r = 0,866), total subtended mass (r = 0,996 and r = 0.990) and perfusion defect mass (r = 0,844 and r = 0,822) (p < 0.01 for all). Details concerning the relevant coronary artery lesion(s), left ventricular mass, subtended mass (per lesion) and perfusion defect (per lesion) are shown in Table 2. The relevant coronary artery lesion(s) define the most proximal lesion of the subsequent vessel with a visual diameter stenosis of ≥ 50%. Left ventricular mass encompasses the mass of the left ventricle automatically calculated using epicardial and endocardial contours. The subtended mass per lesion is the subtended mass calculated by using our Voronoi-based algorithm for a specific lesion. Subsequently total subtended mass can be calculated by adding up the values per lesion. Perfusion defect mass is derived from manual drawing of the perfusion defect. Lastly, the perfusion defect per lesion encompasses the intersection of the perfusion defect and its lesion specific subtended mass. The sum of these lesion specific values encompasses the total mass of the perfusion defect which intersects with the subtended mass of those lesions. The percentage in the last column represents how much of the total (manually drawn) perfusion defect represents the total perfusion defect mass found per lesion.

**Table 1:** Patient characteristics

	N = 33
Male/Female	20 (61%)/13 (39%)
Age (years)	67.8 ± 8.2
Hypertension	4 (12%)
Hyperlipidaemia	17 (52%)
Diabetes mellitus	7 (21%)
Family history of CAD	16 (48%)
Smoking	11 (9%)
Single-vessel disease^a^	16 (49%)
Double-vessel disease^b^	10 (30%)
Triple-vessel disease^c^	7 (21%)

*CAD* Coronary artery disease

^a^Defined as luminal diameter stenosis of ≥ 50% on CCTA in one major epicardial coronary vessel. ^b^Defined as luminal diameter stenosis of ≥ 50% on CCTA in two major epicardial coronary vessels. ^c^Defined as luminal diameter stenosis of ≥ 50% on CCTA in three major epicardial coronary vessels

**Table 2:** Results

Case	Relevant coronary artery lesion(s)	Left ventricular mass (grams)	Subtended mass per lesion (grams)	Perfusion defect mass (grams)	Perfusion defect mass per lesion (grams)	Case	Relevant coronary artery lesion(s)	Left ventricular mass (grams)	Subtended mass per lesion (grams)	Perfusion defect mass (grams)	Perfusion defect mass per lesion (grams)
**1**	mLAD ≥ 70% dRCA ≥ 50%	98	mLAD 22 dRCA 29 Total 51	11	mLAD 10 dRCA 1 Total 11 (100%)	**18**	pLAD ≥ 50% pRCA ≥ 50%	89	pLAD 62 pRCA 24 Total 86	7	pLAD 4 pRCA 3 Total 7 (100%)
**2**	mLAD ≥ 70% D1 ≥ 70% dRCA ≥ 50%	118	mLAD 15 D1 20 dRCA 49 Total 84	3	mLAD 2 D1 0 dRCA 1 Total 3 (100%)	**19**	pLAD ≥ 50% dRCA ≥ 50% Cx ≥ 50%	104	pLAD 39 dRCA 21 Cx 24 Total 84	7	pLAD 4 dRCA 3 Cx 0 Total 7 (100%)
**3**	LM ≥ 50% dRCA ≥ 50%	162	LM 94 dRCA 63 Total 157	12	LM 8 dRCA 4 Total 12 (100%)	**20**	pLAD ≥ 50% Cx ≥ 50%	106	pLAD 48 Cx 35 Total 83	11	pLAD 10 Cx 0 Total 10 (91%)
**4**	pLAD ≥ 50% Cx ≥ 50%	127	pLAD 46 Cx 57 Total 103	6	pLAD 0 Cx 6 Total 6 (100%)	**21**	pLAD ≥ 50%	134	pLAD 45 Total 45	8	pLAD 7 Total 7 (88%)
**5**	dRCA ≥ 50% pLAD ≥ 50% AL ≥ 50%	136	pLAD 42 dRCA 21 AL 30 Total 93	9	pLAD 5 dRCA 2 AL 2 Total 9 (100%)	**22**	pLAD ≥ 50% pRCA ≥ 70% Cx ≥ 50%	94	pLAD 28 pRCA 0 Cx 51 Total 79	7	pLAD 6 pRCA 0 Cx 0 Total 6 (86%)
**6**	pLAD ≥ 50%	220	pLAD 105 Total 105	13	pLAD 13 Total 13 (100%)	**23**	mLAD ≥ 50% pRCA ≥ 50% Cx ≥ 50%	124	mLAD 36 pRCA 14 Cx 31 Total 81	7	mLAD 6 pRCA 0 Cx 0 Total 6 (86%)
**7**	mLAD ≥ 50%	115	mLAD 63 Total 63	5	mLAD 5 Total 5 (100%)	**24**	pLAD ≥ 70% mRCA ≥ 70% MO ≥ 50%	100	pLAD 38 mRCA 0 MO 25 Total 63	12	pLAD 9 mRCA 0 MO 1 Total 10 (83%)
**8**	pLAD ≥ 50% Cx ≥ 50%	73	pLAD 38 Cx 17 Total 55	6	pLAD 6 Cx 0 Total 6 (100%)	**25**	pLAD ≥ 50%	132	pLAD 64 Total 64	8	pLAD 6 Total 6 (75%)
**9**	LM ≥ 50%	67	LM 46 Total 46	5	LM 5 Total 5 (100%)	**26**	mLAD ≥ 50%	139	mLAD 37 Total 37	7	mLAD 5 Total 5 (71%)
**10**	pLAD ≥ 50%	90	pLAD 42 Total 42	5	pLAD 5 Total 5 (100%)	**27**	pLAD ≥ 70%	96	pLAD 43 Total 43	5	pLAD 3 Total 3 (60%)
**11**	pLAD ≥ 50% pRCA ≥ 50% Cx ≥ 50%	90	pLAD 33 pRCA 29 Cx 27 Total 89	10	pLAD 2 pRCA 8 Cx 0 Total 10 (100%)	**28**	mRCA ≥ 50%	145	mRCA 51 Total 51	5	mRCA 3 Total 3 (60%)
**12**	mLAD ≥ 50%	87	mLAD 35 Total 35	10	mLAD 10 Total 10 (100%)	**29**	mLAD ≥ 50%	110	mLAD 39 Total 39	9	mLAD 5 Total 5 (56%)
**13**	mLAD ≥ 70% Cx ≥ 50%	255	mLAD 111 Cx 25 Total 136	8	mLAD 8 Cx 0 Total 8 (100%)	**30**	dLAD ≥ 50% D2 ≥ 50%	163	dLAD 43 D2 25 Total 68	4	dLAD 1 D2 1 Total 2 (50%)
**14**	dLAD ≥ 50% D1 ≥ 70% MO ≥ 50% AL ≥ 50%	98	dLAD 7 D1 30 MO 18 AL 10 Total 65	2	dLAD 0 D1 1 MO 0 AL 1 Total 2 (100%)	**31**	dLAD ≥ 70%	130	dLAD 22 Total 22	3	dLAD 1 Total 1 (33%)
**15**	pLAD ≥ 50% mRCA ≥ 50% Cx ≥ 50%	64	pLAD 45 mRCA 13 Cx 9 Total 67	3	pLAD 1 mRCA 2 Cx 0 Total 3 (100%)	**32**	mLAD ≥ 50% pRCA ≥ 50%	133	mLAD 47 pRCA 0 Total 47	4	mLAD 1 pRCA 0 Total 1 (25%)
**16**	pLAD ≥ 50% pRCA ≥ 50% Cx ≥ 50%	101	pLAD 40 pRCA 28 Cx 28 Total 96	4	pLAD 2 pRCA 2 Cx 0 Total 4 (100%)	**33**	pLAD ≥ 50%	77	pLAD 30 Total 30	5	pLAD 1 Total 1 (20%)
**17**	pLAD ≥ 50% IM ≥ 70%	102	pLAD 46 IM 34 Total 80	3	pLAD 2 IM 1 Total 3 (100%)						

*LM* left main artery, *pLAD* proximal left anterior descending artery, *mLAD* mid left anterior descending artery, *D1* First diagonal branch, *pRCA* Proximal right coronary artery, *dRCA* Distal right coronary artery, *Cx* Circumflex coronary artery, *MO*: Margus Obtusus branch, *AL* Antero lateral branch, *IM* Intermediate branch

## Discussion

In this study we propose a method to fully quantify myocardial perfusion defect mass and subtended myocardial mass at adenosine stress CTP related to the significant coronary artery stenosis at CCTA. Results demonstrate that indeed it seems possible to fully quantify perfusion defects and subtended myocardial mass using a Voronoi-based algorithm allowing for quantitative correlation of coronary artery lesions to corresponding areas of myocardial hypoperfusion.

 Several studies have demonstrated that adding a myocardial perfusion stress test to CCTA improves the diagnostic accuracy for finding hemodynamically significant coronary artery stenoses as compared to a single modality approach. For instance, Magalhaes et al. demonstrated that in a vessel-based analysis, the addition of CTP led to an improvement in the diagnostic accuracy of the combined analysis when compared to coronary CCTA alone (0.79 [95% CI, 0.77–0.82] vs. 0.73 [95% CI, 0.70–0.76], respectively; *P* < 0.0001 for difference). Also, Ko et al. demonstrated that adding CTP to CCTA improved diagnostic accuracy over CCTA alone as the area under the receiver operating curve increased significantly from 0.798 to 0.893 (p = 0,004) on a per vessel-based analysis [5, 6]. Adenosine stress CTP has been shown to be at least as accurate or even superior in the detection of ischemia as compared to single photon emission computed tomography (SPECT) and magnetic resonance imaging (MRI) perfusion. George et al. performed a head to head comparison between CTP and SPECT myocardial perfusion for detecting significant stenoses of 50% or more. It was demonstrated that in the per-vessel analysis, the area under the receiver operating curve of CT perfusion imaging (0.74; 95% CI: 0.71, 0.78) was higher than that of SPECT myocardial perfusion (0.69; 95% CI: 0.66, 0.72) for the diagnosis of a stenosis of at least 50% when considering all vessels (*P* = 0.008) Otton et al. used a perfusion phantom for a direct comparison of the sensitivity of CTP and MRI perfusion in which it was found that the sensitivities of each perfusion modality when directly compared were similar. However, no statistical evidence was given to back this claim [7, 8]. Though nowadays myocardial ischemia on adenosine stress CTP is still assessed semi-quantitatively by visual analysis, quantification of the perfusion defect mass in relation to subtended myocardial mass distal from a significant coronary artery stenosis would be desirable which may help identifying the hemodynamically relevant lesion. As such, Giordano et al. assessed the use of volume of the hypoperfused region calculated from myocardial blood flow at CTP for finding the hemodynamically significant stenosis. They specifically calculated the hypoperfused volume in the myocardial area distal from the stenosis. It was proven that use of the calculated volume had a slightly better accuracy in detecting the hemodynamically significant stenosis as compared to CTP derived myocardial blood flow alone (79% versus 75% respectively) [9]. The Voronoi-based algorithm for calculating subtended mass used on CCTA seems reliable in predicting ischemia on SPECT as is demonstrated in a study by Kurata et al. in which there was a moderate correlation between the summed stress score of SPECT and CCTA based subtended mass as calculated with a Voronoi-based algorithm (r = 0.531 p = 0.001) [10]. The same has been done for MRI perfusion by Fukuyama et al. which showed an even better correlation between Voronoi-based calculation of subtended mass and areas of relative hypoperfusion (r = 0.73 p < 0.001) [11]. Although we were able to apply a Voronoi-based segmentation algorithm on all cases and quantify subtended myocardial mass (per lesion) and perfusion defect mass (per lesion), results from Table 2 show that there was not always agreement between the sum of the myocardial perfusion defect mass per lesion and the total measured myocardial perfusion defect mass. This disagreement can be due to several factors. First of all, only lesions with a visual diameter stenosis of ≥ 50% at CCTA were deemed relevant. However, multiple studies have demonstrated that not only diameter stenosis but also other plaque features contribute to a lesion being hemodynamically significant or not. For instance, Nakazato et al. examined the performance of percent aggregate plaque volume, which represents cumulative plaque volume as a function of total vessel volume by CCTA for identification of ischemic lesions. It was demonstrated that percent aggregate plaque volume provided incremental prediction for lesion ischemia over diameter stenosis (AUC 0.88 [95% CI: 0.78 to 0.99] vs. 0.68 [95% CI: 0.54 to 0.83], respectively; p = 0.02). Also, Yin et al. demonstrated that maximum area stenosis was superior over maximum diameter stenosis in the detection of ischemic lesions (AUC 0.77 versus 0.71 respectively) [12, 13]. Also, in a study by van Rosendael et al. assessing the relationship between lumen area stenosis and myocardial ischemia on CTP it was found that 9% of all vessels showed ischemia even though lumen area stenosis was below 50% [14]. Subsequently, our defined relevant coronary artery lesion at CCTA will not always correspond to the hemodynamically significant lesion causing the perfusion defect. Secondly, we used only the rest myocardial perfusion scan (CCTA) as reference. Therefore, slight discrepancies in contour size, reference points and thus perfusion defect localization may happen. This may lead to a slight mismatch between total perfusion defect mass and perfusion defect mass per lesion. Finally, for manually drawing perfusion defects visual analysis is still needed and may be susceptible to interpretation errors [15].

Von Spiczak et al. introduced a 3D fusion model for combining adenosine stress CTP and CCTA for correlating the ischemic region to the culprit coronary lesion as defined on invasive coronary angiography (ICA). Yet this method remains semi-quantitative and thus only allows for visual assessment of morphology and function [16]. Our method is different as a fully quantitative approach was used allowing not just for intuitive assessment by 3D reconstruction but also for numerical assessment.

Furthermore, a previous study has reported that perfusion territories of coronary arteries vary among individuals. In a per-segment analysis done by Ortiz-Perez et al. 23% of the hyper enhanced regions on cardiac MRI were discordant with the empirically assigned coronary distribution as defined by the standard 17-segment model [17]. Use of Voronoi-based segmentation can overcome this problem as its accuracy has been reported in an animal study using swine hearts in which this method was found to be more accurate than the standard 17-segment model in predicting coronary territories [18, 19].

Results from a study by Dadgar et al. assessing the weight of human hearts demonstrated that weight of the left ventricle varies between 100 and 180 g, which is comparable to our results [20]. Tanabe et al. demonstrated that subtended myocardial volume in combination with subtended CT myocardial blood flow derived from CT myocardial perfusion is a better predictor of obstructive CAD than CT myocardial blood flow alone. A Voronoi-based segmentation algorithm was also used for calculating subtended myocardial volume yielding an average of 42.7 mL for obstructive CAD. Our average subtended mass per lesion (36 g) is only slightly lower when taking into account average density of myocardial tissue of 1055 g/mL to convert mass to volume [21, 22]. This could be due to the fact that contrary to Tanabe no ICA was used for verifying the hemodynamical significance of the relevant coronary artery lesion(s).

## Limitations

This study has several limitations which are innate to its retrospective design and novel nature. Selecting patients with only good or excellent imaging quality on adenosine stress CTP may have introduced selection bias. Consequently, the relatively small number of female patients may have introduced further bias as evidence suggests that females may experience higher myocardial perfusion flow values compared to males [23]. Since the goal of this study was to provide insights into a new proof of principle more testing and further investigation is needed to implement this concept in a larger patient cohort.

## Conclusion

Fully quantifying myocardial perfusion defects and subtended myocardial mass allows to quantitatively correlate coronary artery lesions to corresponding areas of myocardial hypoperfusion at CCTA and adenosine stress CTP. This novel technique may prove especially useful for patients with multivessel disease undergoing invasive coronary angiography as correlation of the perfusion defect and coronary artery lesions gives more insight in myocardial ischemia localization.

## Data Availability

The datasets generated during and analyzed during the current study are available from the corresponding author on reasonable request.

## Code availability

The code generated during and used during the current study is available from the corresponding author on reasonable request.
